# Gimli: open source and high-performance biomedical name recognition

**DOI:** 10.1186/1471-2105-14-54

**Published:** 2013-02-15

**Authors:** David Campos, Sérgio Matos, José Luís Oliveira

**Affiliations:** 1 IEETA/DETI, University of Aveiro, Campus Universitário de Santiago, Aveiro, 3810-193, Portugal

## Abstract

**Background:**

Automatic recognition of biomedical names is an essential task in biomedical information extraction, presenting several complex and unsolved challenges. In recent years, various solutions have been implemented to tackle this problem. However, limitations regarding system characteristics, customization and usability still hinder their wider application outside text mining research.

**Results:**

We present Gimli, an open-source, state-of-the-art tool for automatic recognition of biomedical names. Gimli includes an extended set of implemented and user-selectable features, such as orthographic, morphological, linguistic-based, conjunctions and dictionary-based. A simple and fast method to combine different trained models is also provided. Gimli achieves an F-measure of 87.17% on GENETAG and 72.23% on JNLPBA corpus, significantly outperforming existing open-source solutions.

**Conclusions:**

Gimli is an off-the-shelf, ready to use tool for named-entity recognition, providing trained and optimized models for recognition of biomedical entities from scientific text. It can be used as a command line tool, offering full functionality, including training of new models and customization of the feature set and model parameters through a configuration file. Advanced users can integrate Gimli in their text mining workflows through the provided library, and extend or adapt its functionalities. Based on the underlying system characteristics and functionality, both for final users and developers, and on the reported performance results, we believe that Gimli is a state-of-the-art solution for biomedical NER, contributing to faster and better research in the field. Gimli is freely available at http://bioinformatics.ua.pt/gimli.

## Background

In the biomedical field, a growing amount of data is continuously being produced, resulting largely from the widespread application of high-throughput techniques, such as gene and protein analysis. This growth is accompanied by a corresponding increase of textual information, in the form of articles, books and technical reports. In order to organize and manage these data, several manual curation efforts have been set up to identify, in texts, information regarding entities (e.g., genes and proteins) and their interactions (e.g., protein-protein). The extracted information is stored in structured knowledge resources, such as MEDLINE, Swiss-Prot and GenBank. However, manual annotation of large quantities of data is a very demanding and expensive task, making it difficult to keep these databases up-to-date. These factors have naturally led to increasing interest in the application of Text Mining (TM) systems to help perform those tasks.

One major focus of TM research has been on Named Entity Recognition (NER), a crucial initial step in information extraction, aimed at identifying chunks of text that refer to specific entities of interest. Several NER systems have been developed for the biomedical domain, using different approaches and techniques that can generally be categorized as being based on rules, dictionary matching or Machine Learning (ML). In this study we follow an ML approach, the goal being to train statistical models focused on recognizing specific entity names, using a feature-based representation of the observed data. This presents various advantages over other approaches, such as the recognition of new and short entity names. Moreover, ML solutions have been shown to achieve the best results for this specific domain.

Various techniques for adapting and optimizing ML-based solutions have been proposed in recent years. These can be categorized into one of the following sub-tasks: pre-processing, feature extraction, modelling, and post-processing. In the initial step, the input data is pre-processed to make it readable by computers and to simplify the recognition process. This sub-task is one of the most important, since every single action will affect the entire system behaviour. Tokenisation is a mandatory step, in order to divide natural language texts into discrete and meaningful units. There are several approaches to implement it, depending on the input data and desired output. For instance, Tsuruoka et al. [1] keep words that contain a dash as a single token, while Leaman and Gonzalez [2] create multiple tokens for the same word.

In the feature extraction step, it is important to obtain features that reflect the different characteristics of the sentences and tokens. At the token level, orthographic [2-5] and morphological [1,4,6,7] features are commonly used in order to extract token formation patterns. It is also common to encode domain knowledge as features [2,8] using external resources, such as lexicons of gene and protein names. At the sentence level, linguistic [2,6,9] and local context features [1,3,5,10], such as windows and conjunctions of features, are used to model the links between tokens.

The ultimate goal is to model the observed data using the features extracted in the previous step, thus creating a probabilistic description of the data classes. This task is accomplished using ML models, which can be classified as being supervised or semi-supervised, depending on unannotated data being used or not. Supervised learning, which only uses annotated data, has received most research interest in recent years. Consequently, different supervised models have been used on biomedical NER systems, such as Conditional Random Fields (CRFs) [2,3,9,10], Support Vector Machines (SVMs) [8] and Maximum Entropy Markov Models (MEMMs) [1,5].

Finally, the post-processing stage aims to improve the recognition results, cleaning annotation errors or refining incomplete annotations. The most common methods consist of removing annotations with unmatched parentheses [7,10], adding the results of abbreviation resolution tools [2,8], and extending names using a domain dictionary [10].

Although several open source solutions aimed at recognizing biomedical names have been proposed in recent years, most present one or more of the following limitations: 

•are focused on a specific corpus and/or biomedical domain;

•do not take advantage of state-of-the-art techniques;

•achieved performance results are deprecated and/or not according to similar closed source solutions;

•not configurable and/or easy to use;

•not easily extensible to new features and/or scalable.

In this article we present Gimli, a new open source solution for automatic recognition of biomedical names, namely gene/protein, DNA, RNA, cell type and cell line names. It extends and optimizes the most advanced state-of-the-art techniques in a simple and easy-to-use tool. By default, Gimli already provides high-performance trained models, supporting several known corpora formats. Moreover, it also allows easy and flexible development of new solutions focused on different semantic types, as well as training new ML models with different feature sets and characteristics.

## Implementation

**Figure 1:**
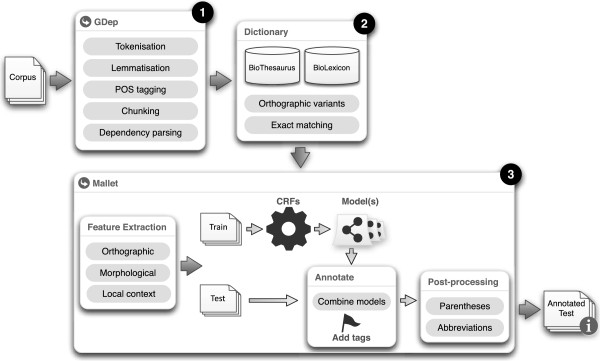
**Overall architecture of Gimli.** Overview of Gimli’s architecture, presenting the workflow of required steps, tools and external resources.

This section presents a detailed description of the resources used and methods implemented, following the workflow of ML-based NER solutions. Figure 1 illustrates Gimli’s architecture, presenting the connections between the various steps.

### Tools and resources

Gimli takes advantage of various publicly available tools and resources. The implementation of Conditional Random Fields for statistical natural language processing is provided by MALLET [11]. GDep [12] is used for tokenization and linguistic processing, namely lemmatization, Part-of-Speech tagging, chunking and dependency parsing. In terms of lexical resources, we use BioThesaurus [13] for gene and protein names, and BioLexicon [14] as the resource for biomedical domain terms.

### Corpora

There are several publicly available corpora that can be used for training and evaluation of NER systems. To allow direct comparison with other tools, we selected two of the most used corpora: GENETAG and JNLPBA. GENETAG [15] is composed of 20000 sentences extracted from MEDLINE abstracts, not being focused on any specific domain. It contains annotations of proteins, DNAs and RNAs (grouped in only one semantic type), which were performed by experts in biochemistry, genetics and molecular biology. This corpus was used in the BioCreative II challenge [16], providing 15000 sentences for training and 5000 sentences for testing. On the other hand, the JNLPBA corpus [17] contains 2404 abstracts extracted from MEDLINE using the MeSH terms “human”, “bloodcell” and “transcription factor”. The manual annotation of these abstracts was based on five classes of the GENIA ontology [18], namely protein, DNA, RNA, cell line, and cell type. This corpus was used in the Bio-Entity Recognition Task in BioNLP/NLPBA 2004 [17], providing 2000 abstracts for training and the remaining 404 abstracts for testing.

Since GENETAG is not focused on any specific biomedical domain, its annotations are more heterogeneous than those of JNLPBA. A brief analysis, considering protein, DNA and RNA classes, shows that GENETAG contains almost 65% of unique entity names, as opposed to the 36% found in JNLPBA.

### Pre-processing

In recent years, various tokenisation solutions have been developed for several domains and languages. Gimli uses the tokeniser from GENIA Tagger [1] (included in Gdep) which is developed for biomedical documents and presents state-of-the-art results in this domain. However, words containing the symbols “/”, “-” or “.” are not always split into multiple tokens. When working at the token level, this may create inconsistencies with the human provided annotations, constraining the model learning process and the recognition of some entity names. For instance, consider that “BRCA-1/2” is taken as one token and that in the gold standard only “BRCA-1” is tagged as an entity name. In the model training phase, the token “BRCA-1/2” as well as its local and contextual features will be considered as a “negative”, which will directly affect the final model. Thus, we decided to make the tokenizer behaviour more consistent, by breaking words containing the symbols “/”, “-” or “.” into multiple tokens.

To train ML models, each token in the training data must be identified as being part, or not, of an entity name. We use the BIO encoding scheme, which is the *de facto* standard. In this scheme tokens are tagged as being at the beginning (tag“B”), inside (tag “I”) or outside (tag “O”) of an entity name.

### Features

Feature extraction is a crucial NER task, since the predictions will be performed based on the information that they encode. Nadeau and Sekine [19] present a complete survey on features used in general NER solutions. Gimli implements a rich set of features, including orthographic, morphological, linguistic parsing, external resources and local context features. We also propose improvements on various features, in order to optimize their behaviour and performance results.

**Figure 2:**
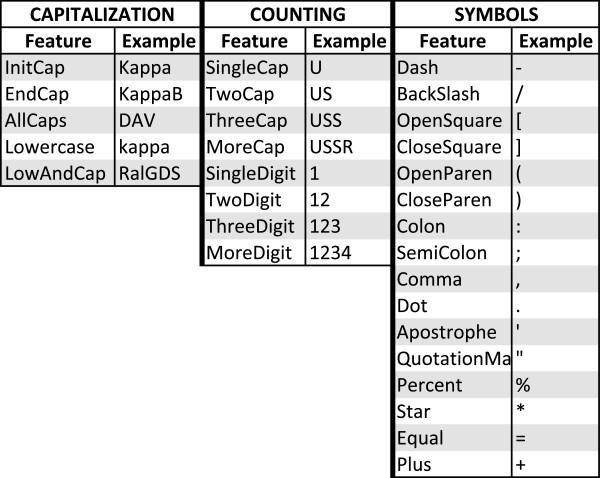
**Orthographic features.** List of orthographic features organized by category.

The purpose of orthographic features is to capture knowledge about word formation. For example, a word that starts with a capital letter could indicate the occurrence of an entity name (e.g., in the protein name “MyoD”). Figure 2 lists the formation patterns used by Gimli to extract orthographic features from tokens.

Morphological features, on the other hand, reflect common structures and/or sub-sequences of characters among several entity names, thus identifying similarities between distinct tokens. To accomplish this goal, three distinct types of morphological features are considered: suffixes and prefixes, char n-grams and word shape patterns. Particular prefixes and suffixes could be used to distinguish entity names. For instance, suffixes like “ase”, “ome” and “gen” frequently occur in gene/protein names [20]. A char n-gram is a subsequence of *n* characters from a given token. This feature type has an identical role to prefixes and suffixes, however it also finds common sub-sequences of characters in the middle of tokens. Finally, it is also important to extract the token’s structure. Collins [21] proposed a method to generate a sequence of characters to reflect how letters and digits are organized in the token. We extended this idea to support symbols too. Thus, three distinct types of word shapes are used by Gimli: 

•Word Shape Type I: replace sequence of digits by “*” (e.g., the structure of “Abc1234” is expressed as “Abc*”);

•Word Shape Type II: replace each letter, digit and symbol by a morphological symbol (e.g., the structure of “Abc:1234” is expressed as “Aaa#1111”).

•Word Shape Type III: replace each sequence of letters, digits and symbols by a morphological symbol (e.g., the structure of “Abc:1234” is expressed as “a#1”).

The most basic internal feature is the token itself. However, in most cases, morphological variants of words have similar semantic interpretations, which can be considered as equivalent. For this reason, lemmatisation is commonly used to group together all inflected forms of a word, so that they can be analysed as a single item. On the other hand, it is also possible to associate each token with a particular grammatical category based on its definition and context, a procedure called Part-of-Speech (POS) tagging. Moreover, we also use chunking, dividing the text into syntactically correlated chunks of words (e.g., noun or verb phrases). The BIO encoding format is used to properly indicate the beginning and end of each chunk. For instance, considering two consecutive tokens that make part of a noun phrase chunk, the tag “B-NP” is associated with the first token and the tag “I-NP” with the second one. In the end, each tag is used as a feature of the respective token.

The previous features provide a local analysis of the sentence. To complement these with information about relations between the tokens of a sentence, we use features derived from dependency parsing. Namely, we follow a strategy similar to the one presented by Vlachos [22], considering only those dependencies that could indicate the presence of an entity name. Thus, we add as features of each token, the lemmas corresponding to each of the following: verbs for which the token acts as subject; verbs for which the token acts as object; nouns for which the token acts as modifier; and the modifiers of that token.

Gimli is further optimized by adding biomedical knowledge to its features. To provide this knowledge, dictionaries of specific domain terms and entity names are matched in the text and the resulting tags are used as features. Thus, the tokens that make part of a matched term contain a feature that reflect such information. For instance, if the term “BRCA” is matched, the feature “LEXICON=PRGE” is added to the token. Two different types of dictionaries are used in Gimli: 

•Gene and protein names: BioThesaurus is the most complete and up-to-date lexical resource for gene and protein names, containing almost 17 million unique names. Due to its size, we decided to filter this lexicon considering only human genes and proteins, obtaining almost 400 thousand unique names. In the end, this lexicon is used to indicate the presence of curated gene and protein names. Since these names could be present in text with small orthographic variations, the matching is performed according the following variation rules, adapted from [23]: 

Replace white spaces per hyphens, and vice-versa;

Remove white spaces and hyphens;

Insert an hyphen on letter-digit sequences;

Replace Roman by Arabic numbers, and Arabic numbers by Greek letters;

Add the prefix “h” and the suffix “p” to acronyms

•Trigger words: specific domain terms may indicate the presence of biomedical names in the surrounding tokens. Instead of using words from training data as proposed in [20], we apply a more general solution, by matching the terms in BioLexicon. This lexical resource contains more than two million relevant biomedical terms, including nouns, verbs, adjectives and adverbs (e.g., “stimulation”, and “activation”).

Higher level relations between tokens and extracted features can be established through windows or conjunctions of features, reflecting the local context of each token. The application of windows consists of adding selected features from preceding and succeeding tokens as features of each token. On the other hand, conjunction of features consists of creating new features by grouping together features of the surrounding tokens. For instance, considering the sentence “Pharmacologic aspects of neonatal hyperbilirubinemia.” and a {-1,1} range of tokens, the following features are added to the token “neonatal”: 

•Windows: the tokens “of” and “hyperbilirubinemia”;

•Conjunctions: the new conjunction feature “of@-1_&_hyperbilirubinemia@1”.

Our tests showed that the best results were obtained using conjunctions. However, Gimli does not use all of the features to generate conjunctions, since this would become impracticable, generating millions of new features. Tsai et al. [9] proposed the use of tokens of the following windows to generate the conjunctions: {-3,-1}, {-2,-1}, {-1,0}, {-1,1} and {0,1}. To improve the context knowledge, we propose a different approach, using lemmas and POS tags instead of tokens, since lemma conjunctions better reflect the pairwise patterns of words, and the POS tags conjunctions provide grammar-based relations and patterns. Following the previous example, instead of the simple token-based conjunction feature, the token “neonatal” now has two conjunction features: POS=IN@-1_&_POS=NN@1 and LEMMA=of@-1_&_LEMMA=hyperbilirubinemia@1. The benefits of these choices were confirmed through various experiments.

### Model

When ML techniques are applied to NER, an algorithm must build a feature and statistical-based representation of target entity names from training data, in order to develop an appropriate response to unseen data. Such methodologies are commonly categorized as being supervised or semi-supervised. Semi-supervised solutions use both annotated and unannotated data, in order to obtain features of the entity names that are not present in the annotated data. Specifically for this task, the usage of unannotated data could contribute to a better abstract learning of the named entities. However, the application of such techniques is computationally heavy and could be implemented as an extension to an equivalent supervised solution. Thus, we decided to follow a supervised training approach, through the application of Conditional Random Fields (CRFs) [24]. Such technique present various advantages over other methods. Firstly, CRFs avoid the label bias problem [24], a weakness of Maximum Entropy Markov Models (MEMMs). Additionally, the conditional nature of CRFs (a discriminative model) relaxes strong independence assumptions required to learn the parameters of a generative model, such as Hidden Markov Models (HMMs) [25]. Finally, Support Vector Machines (SVMs) follow a different approach and have been shown to deliver comparable results to CRFs [26]. However, training complex SVM models for NER may take more time [27,28].

Conditional Random Fields (CRFs) were first introduced by Lafferty et al. [24]. Assuming that we have an input sequence of observations (represented by *X*), and a state variable that needs to be inferred from the given observations (represented by *Y*), a “CRF is a form of undirected graphical model that defines a single log-linear distribution over label sequences (*Y*) given a particular observation sequence (*X*)” [25]. This layout makes it possible to have efficient algorithms to train models, in order to learn conditional distributions between *Y*_*j*_ and feature functions from the observable data. To accomplish this, it is necessary to determine the probability of a given label sequence *Y* given *X*. First, the model assigns a numerical weight to each feature, and then those weights are combined to determine the probability of *Y*_*j*_. Such probability is calculated as follows:

(1) $$p(y|x,\lambda )=\frac{1}{Z(x)}\text{exp}(\underset{j}{\sum }\lambda_{j}F_{j}(y,x)),$$

where *λ*_*j*_ is a parameter to be estimated from training data and indicates the informativeness of the respective feature, *Z*(*x*) is a normalization factor and $F_{j}(y,x)=\overset{n}{\underset{i=1}{\sum }}f_{j}(y_{i-1},y_{i},x,i)$, where each *f*_*j*_(*y*_*i*-1_,*y*_*i*_,*x*,*i*) is either a state function *s*(*y*_*i*-1_,*y*_*i*_,*x*,*i*) or a transition function *t*(*y*_*i*-1_,*y*_*i*_,*x*,*i*) [25].

When considering higher-order models, each label depends on a specific number of *o* previous labels. Thus, the probability will consider not only the previous observation and its features, but *o*-previous observations and features, which better models dependencies and may provide improved results, depending on the target data and task. However, the training complexity of higher-order models increases exponentially with the pre-defined order *o*[29].

### Model combination

The most recent results on biomedical NER clearly indicate that better performance results can be achieved by combining several systems with different characteristics. As an example, the top five systems of the BioCreative II gene mention challenge [16] used ensembles of NER systems, combining distinct models or combining models with dictionary and/or rule-based systems. Additionally, the application of machine learning-based harmonization solutions have been shown to deliver high improvements in terms of performance results [30].

We propose a new and simple combination strategy based on confidence scores. To achieve this, each model provides a confidence value for the annotations predicted for a given sentence. If the models that produced the overlapping annotations predict the same entity class, we follow a straightforward strategy, selecting the annotations from the model that has the highest confidence score and rejecting the predictions of other model(s). On the other hand, if we need to combine annotations of models that predict different entity classes (e.g., as in the JNLPBA corpus), this strategy is extended in order to allow distinct entity types in the same sentence. Thus, instead of selecting a single model to provide the predictions for the entire sentence, this choice is made for each annotation in the sentence. When two or more models provide different annotations for the same chunk of text, we select the annotation given by the model with the highest confidence score. If only one model provides an annotation for a chunk of text, that annotation is accepted.

### Post-processing

In order to solve some errors generated by the CRF model, Gimli integrates a post-processing module that implements parentheses correction and abbreviation resolution. To perform parentheses correction, the number of parentheses (round, square and curly) on each annotation is verified and the annotation is removed if this is an odd number, since it clearly indicates a mistake by the ML model. We also tried to correct the annotations by removing or adding tokens up to the next or previous parenthesis. However, this solution provided worse results than simply removing the annotations.

Regarding abbreviation resolution, we adapt a simple but effective abbreviation definition recognizer [31], which is based on a set of pattern-matching rules to identify abbreviations and their full forms. Such patterns consider some constraints, namely: *a*)the first character of the acronym has to be the first character of the first word in the corresponding long form; *b*) the long form should be longer than the corresponding acronym; and *c*) the long form should not contain the candidate acronym. In the end, we are able to extract both short and long forms of each abbreviation in text. Thus, if one of the forms is annotated as an entity name, the other one is added as a new annotation. Additionally, if one of the forms is not completely annotated, Gimli expands the annotation boundaries using the result from the abbreviation extraction tool.

## Results and discussion

To analyse the impact of various techniques and compare the final results with other existing solutions, we use common evaluation metrics: Precision (i.e., positive predictive value) the ability of a system to present only relevant items; Recall (i.e., sensitivity) the ability of a system to present all relevant items; and F-measure, the harmonic mean of precision and recall. These measures are formulated as follows:

(2) $$\;P=\frac{\text{TP}}{\text{TP}+\text{FP}},\;R=\frac{\text{TP}}{\text{TP}+\text{FN}},\;F1=2\frac{\mathrm{P.R}}{P+R},$$

where TP is the amount of true positives, FP the number of false positives and FN the amount of false negatives.

### Preliminary experiments

During the development of Gimli, various optimizations and decisions had to be performed to achieve the best possible results. In order to run such experiments, we randomly split the training part of each corpus into training and development sets, using 80% of the data for training and the remaining 20% for development testing. Accordingly, from the 15000 sentences of the training part of GENETAG, 12000 sentences are used for training and 3000 sentences for development testing. Regarding JNLPBA, considering the 2000 training abstracts, we now use 1600 abstracts for training and the remaining 400 abstracts for development testing. Most experiments on the development stage, namely tokenization and feature set optimization, were performed using first-order CRF models with forward (left to right) text parsing.

#### Tokenization

To evaluate the impact of the tokenization changes introduced in Gimli, we compared the results achieved against the use of the original tokenization. This analysis only applies to the GENETAG corpus, since JNLPBA is provided as tokenized text. Using the development set, an improvement of 8.28% in F-measure was achieved when applying a model trained on tokens provided by our proposed tokenization as compared to using the original version of GENIA Tagger. When applied to the final test set, and considering the alternative annotations provided, the improvement in F-measure is 2.53%. Such results clearly show the positive contribution of our tokenization approach on Gimli.

#### Feature set

**Figure 3:**
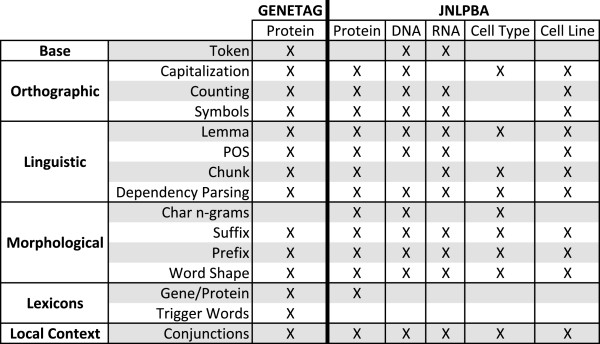
**Feature set per corpus and entity.** Feature set applied to each corpus and entity type. Features marked with an “X” are used in the final feature set for that entity type.

Each feature encodes specific characteristics of target annotations, providing a different contribution in the learning process. In order to evaluate their impact in the recognition performance, we initially grouped features that encode similar information into logical sub-classes for each feature type, as shown in Figure 3. We then followed a backward elimination approach to find the best feature set for each entity type, by removing each sub-class from the complete feature set and analysing its impact in the results. Although small improvements or drops may not be significant regarding performance improvements, they indicate that adding or removing a specific feature may have an impact on the final performance results, which is relevant when considering the inclusion (or not) of that feature. When such cases occurred, we decided to keep the feature when a small improvement occurred and remove it when a small drop is present. In the end, the features that presented a negative impact when removed from the initial set were included in the final feature set, as indicated in Figure 3. For instance, our trigger words approach provides a slight positive impact in the recognition of gene and protein names in GENETAG, resulting in an F-measure improvement of 0.11%. However, a negative impact is observed on JNLPBA, with a 0.39% decrease of F-measure. We believe that the obtained results are a consequence of the corpus specificity, since BioLexicon terms may point to the presence of entity names that were not considered in the specific corpus and/or entity type.

This approach helps experts to better understand the linguistic characteristics of each entity type on each corpus. Thus, the final feature sets seem to reflect the complexity and heterogeneity associated with each entity type and corpus. For instance, the absence of the original tokens for protein, cell line and cell type on JNLPBA may indicate less heterogeneity, as the use of lemmatization appears to better reflect and generalize the target names. Overall, the feature set required by GENETAG is more complex than the ones used on JNLPBA, discarding the original tokens and some orthographic and morphological features. This is consistent with the idea that the entity names present on GENETAG are more heterogeneous than those present on JNLPBA, as suggested before.

#### Conjunctions VS Windows

Local context, as encoded in windows or conjunctions of features, has a great impact in recognition performance. We therefore analysed in detail the impact of using these two alternatives, considering basic and improved solutions. Thus, four different configurations are considered in our analysis: 

•Token conjunctions: form conjunctions as the concatenation of tokens taken from the following windows {-3,-1}, {-2,-1}, {-1,0}, {-1,1} and {0,1};

•Optimized conjunctions: the same windows as the previous configuration but using lemmas and POS tags for the conjunctions, instead of tokens;

•Windows tokens: use each token from the window {-2,2};

•Windows optimized: use lemmas, lexicon matching, biomedical concepts matching and tokens in the window {-3,3}, and all the features in the window {-1,1}.

**Figure 4:**
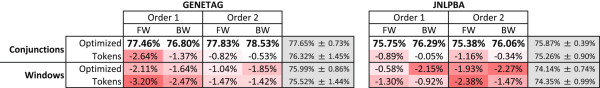
**Comparison of windows and conjunctions results on development sets.** Comparison of F-measure results achieved by token-based and optimized windows and conjunctions in the development sets of both corpora, considering exact matching evaluation, different model orders and text parsing directions. Results for the JNLPBA corpus indicate the overall performance, i.e. across entity types. FW: Forward, and BW:Backward.

Figure 4 presents the performance (F-measure) achieved with the four approaches. Results are shown for CRF models of order 1 and 2 with forward and backward parsing directions, as explained in the next section. Optimized conjunctions present the best results on both corpora, considerably outperforming conjunctions with tokens. Conjunctions of features seem to perform better than windows for this task, as indicated by the fact that using simple token conjunctions provided better results than even the optimized windows of features. Interestingly, while the optimized windows present better results than windows with tokens on GENETAG, in the case of JNLPBA using just the tokens provides better results for the models trained with backward parsing direction. Overall, optimized conjunctions present the most constant behaviour, presenting the best results and less deviation. On the other hand, using tokens resulted in higher deviation on both approaches.

This analysis indicates that choosing the right method to encode local context is fundamental, since an untidy decision may deliver considerably worse results. As we can see, the average F-measure differences between the best and worst solutions on GENETAG and JNLPBA are of 2.13% and 1.73%, respectively.

### Model combination analysis

The usual direction to parse a text is from left to right (forward). However, previous studies [10,32] have shown that parsing the text from right to left (backward) may provide better results, which has been shown to be a consequence of the asymmetric implementation of CRF models in MALLET [32]. Additionally, we believe that using CRFs with different orders will extract different context based characteristics from text. Thus, we decided to train first and second order CRF models, considering both forward and backward text parsing.

**Figure 5:**
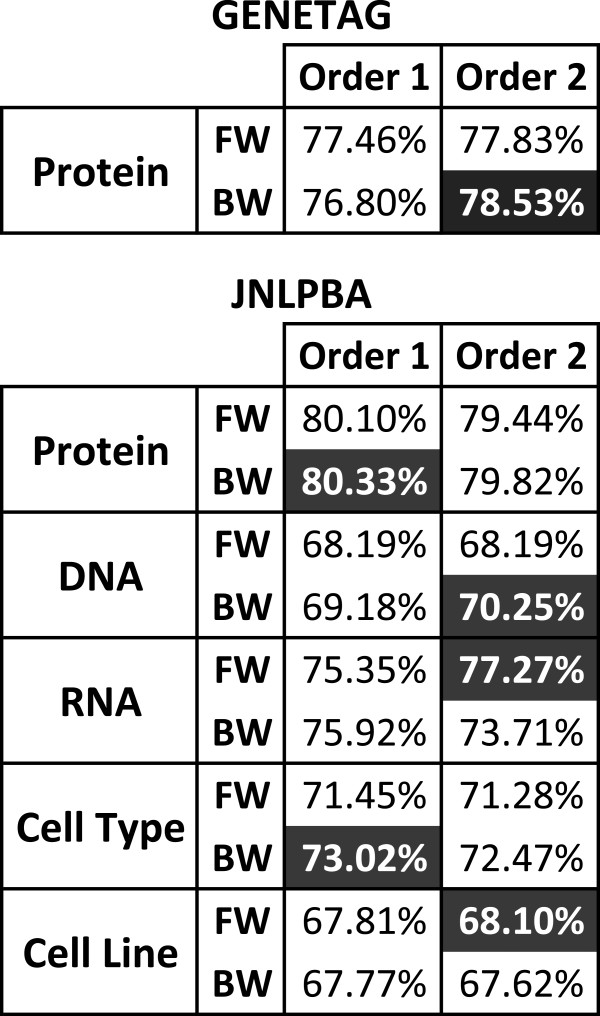
**Preliminary results on development sets.** Preliminary F-measure results achieved by Gimli in the development sets of both corpora, considering exact matching evaluation, different model orders and text parsing directions. The best combination of model order and parsing direction for each entity type is highlighted. FW: Forward, and BW:Backward.

Initial evaluation results on GENETAG and JNLPBA are presented in Figure 5, using the previously selected feature set (Figure 3). As we can see, the application of different CRF orders and parsing directions provides significant performance differences. For instance, considering RNA on JNLPBA, the difference between different parsing directions is above 3%, and the difference between different CRF orders is approximately 2%. Overall, backward models present the best results, which confirms the benefit of using backward text parsing. Moreover, due to the different entity names’ heterogeneity existent in both corpora, different model orders are required. On GENETAG, the best results are achieved using second order models. On the other hand, the best results for protein and cell type on JNLPBA are achieved using first order models.

**Figure 6:**
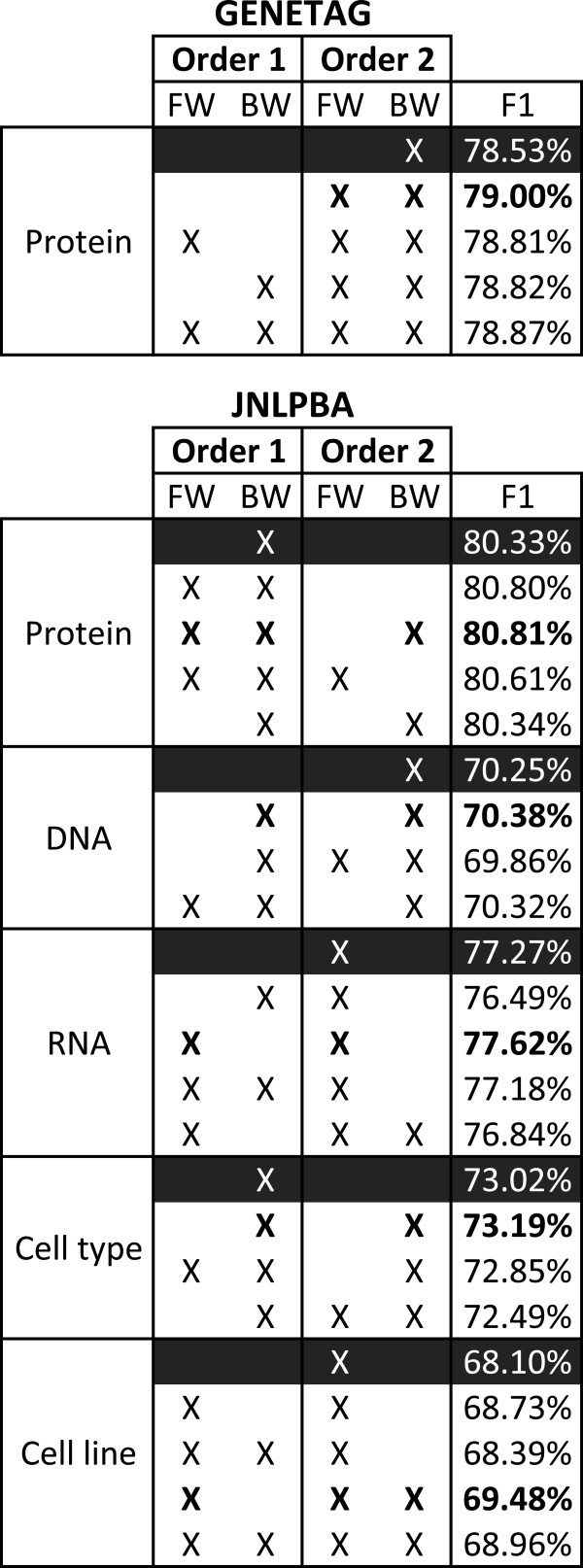
**Combination results on development sets.** F-measure results achieved for each class with the combination of several models in the development sets of both corpora, considering exact matching evaluation. The combination results are compared with the best performing model obtained in previous experiments (Figure 5). FW: Forward, BW:Backward, and F1: F-measure.

To combine the various models for each class on each corpus, we performed a sequential analysis of the combination results. Thus, we first combined the two best models for each class and, if the performance was better than the best model alone, we kept adding models to the two best, in order to find the best set. If the combination result of the two best models was not better than the best model, we tried combining the best model with others, until a better combination was obtained. If the combination did not improve the results, only the model with the best result was used. Figure 6 presents the results of our analysis. Even with the simple combination approach used by Gimli, the harmonisation strategy improves the best model results. An average improvement of 0.5% is verified. Overall, the best combination results are achieved by combining the two best performing models. Moreover, models with low performance results also contribute to a better model combination, by providing heterogeneity that is not present in other models. For instance, on cell line the best model combination is achieved by adding the worst performing model.

**Figure 7:**
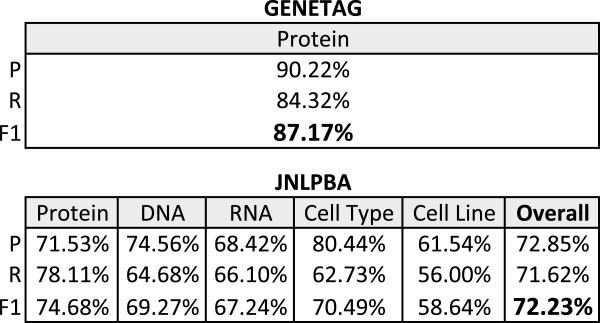
**Final results.** Final Precision (P), Recall (R) and F-measure (F1) results achieved by Gimli on test data of both corpora.

Figure 7 presents the final results achieved on both corpora, considering the final and unseen test data of both corpora. Note that the evaluation strategies of the two challenges are slightly different. On JNLPBA only full matches are considered correct, requiring both left and right boundaries to match exactly. On the other hand, GENETAG evaluation allows minor mistakes, based on alternative names that were previously accepted by human annotators during the preparation of the corpus.

### Feature contributions

**Figure 8:**
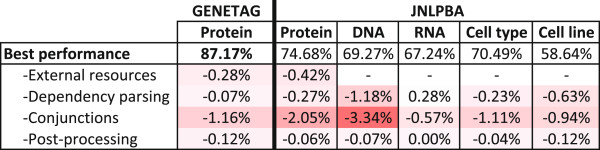
**Key features’ contribution.** F-measure contribution of key features on GENETAG and JNLPBA considering all semantic types.

In order to evaluate the overall contribution of some high-end features implemented by Gimli, we performed an analysis on both corpora, considering the removal of such features from the best feature set for each entity type. Figure 8 presents the observed differences, reflecting the features’ contribution. Overall, removing conjunctions causes the highest negative impact, considerably reducing the performance results. Dependency parsing also contributes positively to the final results, namely on DNA and cell line. On the other hand, removing dependency parsing features from RNA improves the results. However, this is a consequence of the algorithm to combine the models of different entity types. When evaluated alone, RNA recognition presents an F-measure of 68.97%. Removing dependency parsing features, this value drops slightly to 68.91%, reflecting the positive contribution of such features. As expected, lexicons also provide a positive contribution, increasing the models’ precision. Post-processing, on the other hand, introduces just a small positive contribution. For instance, on RNA, the absence of post-processing methods does not affect the performance in any way.

### Performance analysis

**Figure 9:**
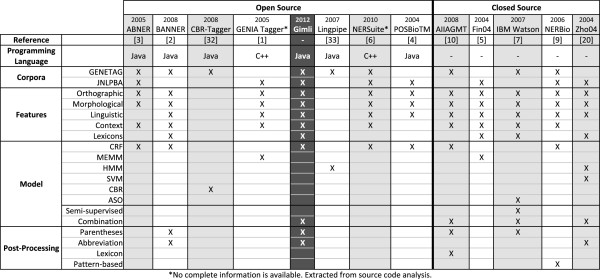
**Biomedical NER systems overview.** Summary of the open and closed source systems’ characteristics, presenting the used programming languages, features, models and post-processing techniques. CBR-Tagger [33] and Lingpipe [34] were also included in this analysis.

To evaluate Gimli and understand its behaviour in comparison with existing solutions, we collected the best open and closed source systems for biomedical named entity recognition. Figure 9 presents a summary description of the systems’ characteristics, comparing them against Gimli. Overall, we collected a total of 12 systems, where seven are open source and five closed source. Our study of these systems allowed to identify some current trends of biomedical NER systems: 

•The most used ML model is CRF (6 systems);

•Almost all the discriminative ML models use orthographic, morphological and basic-linguistic (POS tags and lemmas) features;

•Only 3 systems use model combination, all of which are closed source;

•Only 5 systems use post-processing techniques, where 4 are closed source.

•8 systems support GENETAG and 6 systems support JNLPBA;

•Only 3 systems support both corpora, where 2 are open source;

Based on these facts, we can argue that closed source solutions are commonly developed for a specific corpus, being focused on only one specific goal. However, those solutions present the most advanced techniques. On the contrary, open source solutions do not always take advantage of high-end techniques.

**Figure 10:**
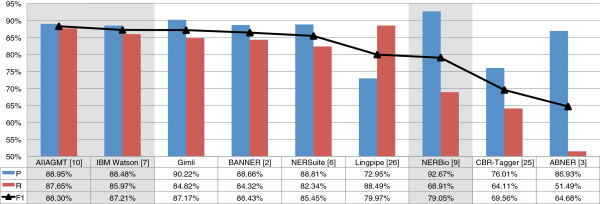
**Results comparison on GENETAG corpus.** Comparison of the Precision (P), Recall (R) and F-measure (F1) results achieved by Gimli on GENETAG corpus, comparing with both open and closed source solutions. Results of closed source solutions are shown with a shaded background.

**Figure 11:**
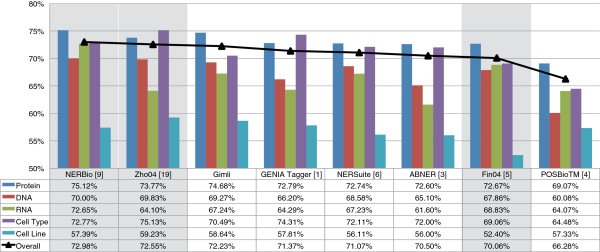
**Results comparison on JNLPBA corpus.** Comparison of the F-measure results achieved by Gimli on JNLPBA corpus, comparing with both open and closed source solutions. The overall result reflects the achieved performance considering the five entity types, and the results of closed source solutions are shown with a shaded background.

Figures 10 and 11 present the results obtained on GENETAG and JNLPBA corpus respectively, comparing Gimli against open and closed source systems. On the GENETAG corpus, Gimli outperforms all the open source solutions, achieving an F-measure of 87.17%. It presents a significant improvement of 0.74% over the second best system, BANNER. In comparison with NERSuite, Gimli presents an improvement of 1.72%. Overall, it presents the best results both on precision and recall. Regarding closed source solutions, Gimli presents the third best result, with a similar performance as the winner of the BioCreative II Gene Mention challenge [16] (IBM Watson), which uses semi-supervised ML and forward and backward model combination. Overall, AIIAGMT presents the best result on this corpus (with 88.30% of F-measure). However, the presented solution was prepared specifically for this corpus, applying a complex combination strategy that requires eight different CRF models using two different CRF frameworks.

Considering the JNLPBA corpus, Gimli outperforms all the open source solutions, achieving an overall F-measure of 72.23%. It presents a significant improvement of F-measure in comparison with the second best system, GENIA Tagger. Compared to the best java-based solution (ABNER), Gimli presents an improvement of 1.73% of F-measure. It considerably outperforms open source systems in recognition of protein, DNA, RNA and cell line names. However, it is outperformed in the recognition of cell types. Regarding closed source solutions, Gimli presents the third best result, with similar results as the winner of the NLPBA Shared Task [17] (Zho04). When compared with the second best participant of this challenge (Fin04), Gimli presents an overall improvement of 2.17% of F-measure. NERBio, the best system on this corpus, implements a rule-based post-processing method that was prepared specifically for this corpus. Moreover, NERBio presents a very low performance result (79.05% of F-measure) on GENETAG, which could indicate some limitations in adapting this solution to different corpora.

Considering a non-blind model combination strategy, as taken by Hsu et al. [10], Gimli presents slightly better results, achieving an F-measure of 87.36% on GENETAG and 72.69% on JNLPBA. Such results outperform all the systems that participated on both challenges.

Overall, Gimli significantly outperforms all the existent open source solutions on both GENETAG and JNLPBA, by simply adapting the feature set used for each corpora and entity type. Moreover, it also presents competitive results when compared with similar closed source solutions for both corpora.

### Speed analysis

The various experiments to check training and tagging speed were performed in a machine with 8 processing cores @ 2.67 GHz and 16GB of RAM.

The training speed varies with the corpus size, feature set complexity and model order. Considering the training parts of both corpora and the final feature set, a second-order CRF model takes on average one hour to be trained. On the other hand, a first-order CRF model requires on average 30 minutes.

In order to check tagging speed and Gimli tractability, we developed a simple algorithm to annotate MEDLINE abstracts using multi-threading processing. This solution includes input XML parsing, sentence splitting, Gimli integration and output generation in XML. It uses a single second-order CRF model, but model combination can be easily integrated with reduced speed impact, taking advantage of multi-threaded processing. During this analysis, we considered various configurations of Gimli, enabling and disabling the most resource expensive techniques. Thus, if users prioritize annotation speed over high performance results, windows can be used instead of conjunctions and dependency parsing can be removed from the workflow. Moreover, in order to use the available resources as much as possible, the number of running threads must be inversely proportional to the complexity of the used techniques, since complex techniques require more processing resources. The following results were obtained: 

•Conjunctions with dependency parsing: 4 threads, 20 sentences/second;

•Conjunctions without dependency parsing: 6 threads, 86 sentences/second;

•Windows without dependency parsing: 8 threads, 232 sentences/second.

## Conclusions

This article presents Gimli, a new open source and high-performance solution for biomedical named entity recognition on scientific documents, supporting the automatic recognition of gene/protein, DNA, RNA, cell line and cell type names. Gimli implements a machine learning-based solution, taking advantage of Conditional Random Fields. Moreover, it supports a rich set of features, including orthographic, morphological, linguistic-based and also domain knowledge features, through the implementation of a lexicon matching technique. Additionally, Gimli implements advanced conjunctions of features, creating new features based on windows of lemmas and part-of-speech tags. In order to correct mistakes generated by the CRF models, Gimli also integrates a post-processing module, implementing parentheses correction and abbreviation resolution, aimed at extending incompletely tagged names. Finally, Gimli also combines several forward and backward models to achieve the best results.

In order to evaluate Gimli and compare it against existing systems, we used two well-known corpora: GENETAG and JNLPBA. In the end, it achieved F-measure results of 87.17% and 72.23% on each corpora, respectively. These results were compared to the systems that participated in the challenges where the corpora were used, BioCreative II Gene Mention and NLPBA Shared Task. Gimli outperforms all existing open source solutions on both corpora, presenting significant improvements both in results and techniques used.

Gimli is an off-the-shelf solution that can be used through two different endpoints, thinking on users with different goals and expertise: 

•Command Line Interface (CLI): automatic scripts with easy access to main functionalities, allowing the annotation of documents using provided models, and training new models focused on different entity types, using a configuration file to customize the feature set and model parameters;

•Application Programming Interface (API): provides complete access to implemented features and associated infrastructure, allowing the easy integration of Gimli in complex text mining workflows, by using, extending and/or adapting the provided functionalities.

Overall, we believe that Gimli provides various characteristics that make it a state-of-the-art solution for biomedical NER: 

•High-end techniques: Gimli applies various state-of-the-art techniques and proposes optimizations on various methods, presenting innovative and high-performance alternatives. Moreover, it integrates various solutions that are only present on closed source solutions, such as dependency parsing, chunking and model combination;

•Flexible: Gimli was built thinking on flexibility, founded on a strong infrastructure that allows adding new features and extending or changing existing ones. Moreover, Gimli offers the only CLI that allows feature set and model parameters definition;

•Scalable: the internal infrastructure is ready to scale, supporting the development of more complex solutions. Moreover, Gimli is ready to be used on multi-threaded applications, in order to process millions of documents;

•Documentation: we provide complete and detailed documentation of Gimli, in order to use both CLI and API. Together with the associated simplicity and self-explanatory code, we believe that Gimli is easy to use, change and extend.

Developers and researchers of the biomedical domain, especially text mining experts, can take advantage of the presented characteristics to develop their own NER and/or post-NER applications. Gimli reduces the required effort to develop innovative NER solutions, increasing the users’ time to focus on their main goals. Thus, it can be used to support the development of various multi-disciplinary solutions: *a*) NER using different corpora and target entity names, such as disorders and chemicals; *b*) normalization; *c*) relation extraction, such as protein-protein interactions; and *d*) information retrieval.

With the results achieved and the characteristics presented by our system, we strongly believe that Gimli is a state-of-the art solution for biomedical named entity recognition, contributing to faster and better research in the field.

### Future Work

Although Gimli already incorporates various improvements on existing tools, some aspects can be further explored. We are currently investigating other approaches for model combination, considering for example the introduction of domain knowledge information and/or context based harmonisation through the use of dictionaries or machine learning-based solutions [30]. As for the recognition of particular entity types such as DNA, RNA, cell type and cell line, we are working on improving the lexicons in order to achieve better precision. An interesting area to explore is the use of feature induction [35] to automatically extract informative features from texts, in order to improve the feature set and obtain “hidden” characteristics of the tokens. The second technique that could be studied is semi-supervised learning [36], using both annotated and unannotated data in order to extract characteristics of the unlabelled data that could contribute to better recognition of entity name boundaries. Regarding the use of Gimli, it could be interesting to implement a set of web services to streamline its integration in other tools and disseminate the simple and fast annotation of scientific documents. Furthermore, although Gimli offers a simple to use command-line application, developing a GUI interface could simplify the analysis of the generated annotations.

There are already various solutions being developed on top of Gimli, such as: *a*) a framework for biomedical information extraction supporting ML and dictionary-based methodologies for normalization of biomedical concepts; *b*) a solution based on semi-supervised NER for gene and protein names recognition; and *c*) an information retrieval solution for knowledge discovery focused on degenerative diseases.

## Availability and requirements

•**Project name:** Gimli

•**Project home page:**http://bioinformatics.ua.pt/gimli

•**Operating system(s):** Platform independent

•**Programming language:** Java

•**Other requirements:** Java 1.6 or higher

•**License:** Creative Commons Attribution-NonCommercial-ShareAlike 3.0 Unported License

•**Any restrictions to use by non-academics:** Non-commercial use

## Abbreviations

CRFs: Conditional Random Fields; HMMs: Hidden Markov Models; MEMMs: Maximum Entropy Markov Models; ML: Machine Learning; NER: Named Entity Recognition; POS: Part-of-Speech; SVMs: Support Vector Machines

## Competing interests

The authors declare that they have no competing interests.

## Authors’ contributions

DC participated in the design and implementation of the system and drafted the manuscript. SM and JLO conceived the study, participated in its design and coordination and helped to draft the manuscript. All authors read and approved the final manuscript.
